# Single cell epigenomic and transcriptomic analysis uncovers potential transcription factors regulating mitotic/meiotic switch

**DOI:** 10.1038/s41419-023-05671-w

**Published:** 2023-02-17

**Authors:** Fa-Li Zhang, Yan-Qin Feng, Jing-Ya Wang, Ke-Xin Zhu, Lu Wang, Jia-Mao Yan, Xiu-Xiu Li, Jun-Jie Wang, Wei Ge, Massimo De Felici, Wei Shen

**Affiliations:** 1 College of Life Sciences, Qingdao Agricultural University, Qingdao, 266109 China; 2 Department of Biomedicine and Prevention, University of Rome Tor Vergata, Rome, 00133 Italy

**Keywords:** Oogenesis, Cell biology

## Abstract

In order to reveal the complex mechanism governing the mitotic/meiotic switch in female germ cells at epigenomic and genomic levels, we examined the chromatin accessibility (scATAC-seq) and the transcriptional dynamics (scRNA-seq) in germ cells of mouse embryonic ovary between E11.5 to 13.5 at single-cell resolution. Adopting a strict transcription factors (TFs) screening framework that makes it easier to understand the single-cell chromatin signature and a TF interaction algorithm that integrates the transcript levels, chromatin accessibility, and motif scores, we identified 14 TFs potentially regulating the mitotic/meiotic switch, including TCFL5, E2F1, E2F2, E2F6, E2F8, BATF3, SP1, FOS, FOXN3, VEZF1, GBX2, CEBPG, JUND, and TFDP1. Focusing on TCFL5, we constructed *Tcfl5*^+/−^ mice which showed significantly reduced fertility and found that decreasing TCFL5 expression in cultured E12.5 ovaries by RNAi impaired meiotic progression from leptotene to zygotene. Bioinformatics analysis of published results of the embryonic germ cell transcriptome and the finding that in these cells central meiotic genes (*Stra8*, *Tcfl5*, *Sycp3*, and *E2f2*) possess open chromatin status already at the mitotic stage together with other features of TCFL5 (potential capability to interact with core TFs and activate meiotic genes, its progressive activation after preleptotene, binding sites in the promoter region of *E2f2* and *Sycp3*), indicated extensive amplification of transcriptional programs associated to mitotic/meiotic switch with an important contribution of TCFL5. We conclude that the identified TFs, are involved in various stages of the mitotic/meiotic switch in female germ cells, TCFL5 primarily in meiotic progression. Further investigation on these factors might give a significant contribution to unravel the molecular mechanisms of this fundamental process of oogenesis and provide clues about pathologies in women such as primary ovarian insufficiency (POI) due at least in part to meiotic defects.

## Introduction

The mechanisms underlying the transition from the mitotic to the meiotic cycle that occurs in germ cells at the beginning of meiosis have been poorly characterized in female mammals [1–3]. STRA8 has been proposed as the main molecular effector of the pro-meiotic action of retinoic acid (RA) both in male and female germ cells [4]. Recently, it has been demonstrated that STRA8 directly upregulates a large set of genes by binding to their promoter in male germ cells at the preleptotene stage [5]. STRA8 is, however, not sufficient to induce meiotic entry by itself, the germ cells must also be in a competent state [6–8]. Moreover, entering meiosis requires the expression of at least two other factors, namely DAZL and DMRT1, that appear to be upstream of *Stra8*. The RNA binding protein DAZL is expressed in both XX and XY mouse germ cells at the time of arrival in the gonadal ridges. *Dazl-*deficient mice are infertile because of germ cell-differentiation defects. In *Dazl-*deficient ovaries, the expression of genes normally expressed in meiotic cells including *Stra8*, is not detectable, a finding considered as an indication that DAZL is necessary to define a “meiosis-competent” germ cell [9]. DMRT1 has been suggested as a transcriptional activator of *Stra8* itself in female mouse germ cells it is [10]. Despite many studies, the underlying regulatory mechanisms of the mitosis/meiosis switch especially concerning the transcription factors (TFs) involved, remain largely unknown. Progress in knowledge about these mechanisms is important not only to reveal the biology of this fundamental process of oogenesis but also for understanding the etiology of some fertility dysfunctions such for example primary ovarian insufficiency (POI). POI, affecting approximately 2% of women worldwide, is an ovarian dysfunction associated with premature ovarian aging and characterized by amenorrhea before age 40 [11]. POI is a highly heterogeneous disease involving many factors, including genetic factors, autoimmunity, and idiopathic factors [12]. About 20-25% of cases are thought to be genetically linked, with mutations in multiple genes associated with gonadal development, meiosis, and DNA repair, such as NOBOX, FIGLA, BMP15, and GDF9 [13–16]. Several studies in mice have shown that abnormal mitotic/meiotic switch results in the meiotic arrest and oocyte degeneration, ultimately leading to POI formation [12].

To investigate the TF regulatory network of mitotic/meiotic switch, in the present study, we investigated changes in chromatin accessibility and gene transcripts by combining scATAC-seq and scRNA-seq. Notably, we developed a rigorous screening framework to identify TFs that potentially regulate mitotic/meiotic switches.

## Results

### Single-cell profile of the epigenome and transcriptome of the ovarian cell populations

**Fig. 1:**
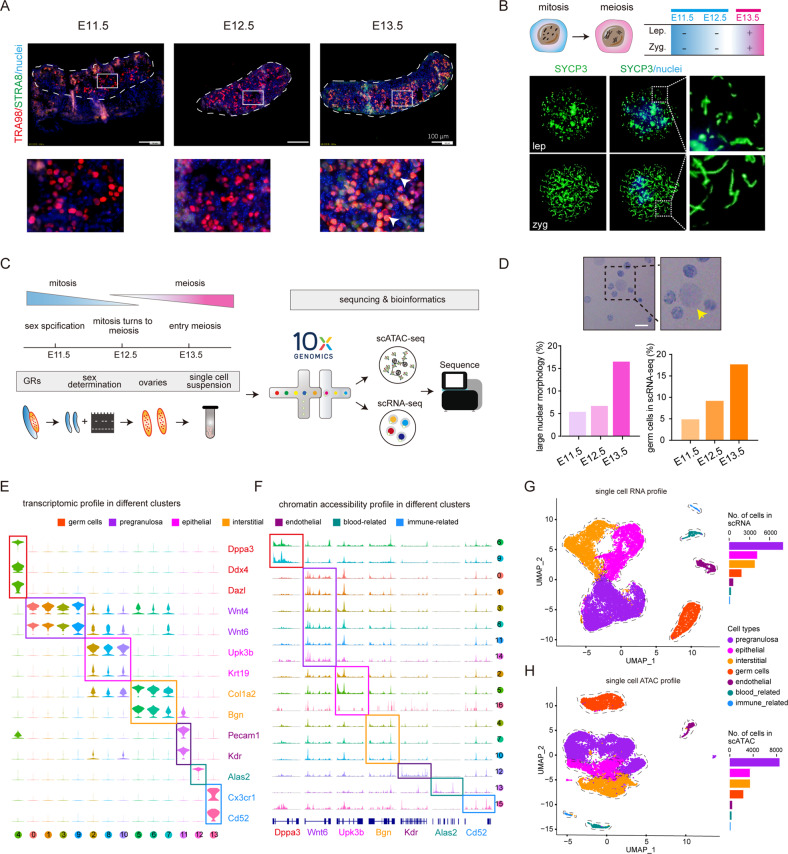
Single-cell atlas of E11.5–13.5 mouse ovaries. **A** E11.5–13.5 female mouse genital ridge sections were stained as indicated. Enlarged images are shown on the bottom; Red shows TRA98, green shows STRA8, and blue shows nuclei; bar = 100 μm. **B** Identification of the time of meiotic initiation according to chromosome cytospreads; Green shows SYCP3 and blue shows nuclei; bar = 1 μm. **C** Workflow employed to investigate the molecular mechanism of meiotic initiation by single-cell multiomics. GRs gonadal ridges. **D** Example of normal and large nuclei (up). Percent of germ cells with a large nucleus and of germ cells identified in the gonadal cell populations using scRNA-seq; bar = 10 μm. **E** Violin plot of marker gene expression across seven ovarian cell types, including germ cells, epithelial cells, endothelial cells, pregranulosa cells, interstitial cells, immune-related cells, and blood-related cells. **F** Chromatin accessibility of key marker genes in the different clusters. **G**, **H** Single-cell RNA profile (**G**) and single-cell ATAC profile (**H**) of ovarian cells of E11.5–13.5 female mouse genital ridge; The barplot on the right shows the number of cells in different cell types.

Since the expression of STRA8 is a crucial marker for meiotic initiation [17], to precisely identify the meiotic initiation timing, we observed the female gonadal ridges/ovaries of E11.5–13.5 for this protein. According to the previous study [17, 18], we found no obvious STRA8 positive cells at E11.5-12.5, while at E13.5, positive germ cells were clearly observed (Fig. 1A). Likewise, SYCP3 immunofluorescence of germ cell cytospreads showed the first morphological signs of the initiation of meiosis in ovaries between E12.5 and E13.5 (Fig. 1B). Having established this timing basis, we next designed a framework that included the study of chromatin accessibility (scATAC-seq) and transcriptional changes (scRNA-seq) from E11.5 up to E13.5 female gonads (Fig. 1C).

As noted, in preparations for the scATAC-seq library, we observed nuclei of distinct size (Fig. 1D), attributable to germ cells, undergoing dynamic changes in chromatin organization as reported by others [19]. Notably, the percent of large nuclei was very similar to that of germ cells identified in the scRNA-seq analyses (Fig. 1D).

To ensure the accuracy of downstream data analysis, we adopted a strict quality control strategy both for scATAC-seq and scRNA-seq. Using such stringency, we analyzed 18,444 single cells for chromatin accessibility information and 18,466 cells for scRNA-seq analysis (Supplementary Fig. S1A–D).

On the basis of scRNA-seq and according to the expression of specific marker genes, cells were divided into seven cell types as follows: germ cells (1798 cells with markers *Dppa3*, *Ddx4*, and *Dazl*) [20–23], pregranulosa cells (7870 cells with markers *Wnt4* and *Wnt6*) [24–26], epithelial cells (4094 cells with markers *Upk3b* and *Krt19*) [27, 28], interstitial cells (3734 cells with markers *Col1a2* and *Bgn*) [29, 30], endothelial cells (585 cells with markers *Pecam1* and *Kdr*) [31, 32], blood-related cells (264 cells with markers *Alas2*) [33], and immune-related cells (121 cells with markers *Cx3cr1* and *Cd52*) [34, 35] (Fig. 1E, G). These were next allocated into 14 clusters (Supplementary Fig. S2A).

For scATAC-seq, seven cell types were also identified (2138 germ cells, 8787 pregranulosa cells, 3517 epithelial cells, 3265 interstitial cells, 355 endothelial cells, 275 blood-related cells, and 107 immune-related cells), and allocated into 17 clusters (Fig. 1H and Supplementary Fig. S2B), demonstrating distinct promoter chromatin accessibility of key marker genes for cell types in different clusters (Fig. 1F). Moreover, the scATAC and scRNA status of the ovarian cell populations at each developmental age were analyzed using the UMAP algorithm. As expected, each cell type was differently distributed and changed dynamically in function during the succession of developmental stages (Supplementary Fig. S2C, D). On the whole, the UMAP profiles of scATAC status at differing time points, such as the distribution of different cell types at different points in time, were highly consistent with various developmental stages (Supplementary Fig. S2E, F).

**Fig. 2:**
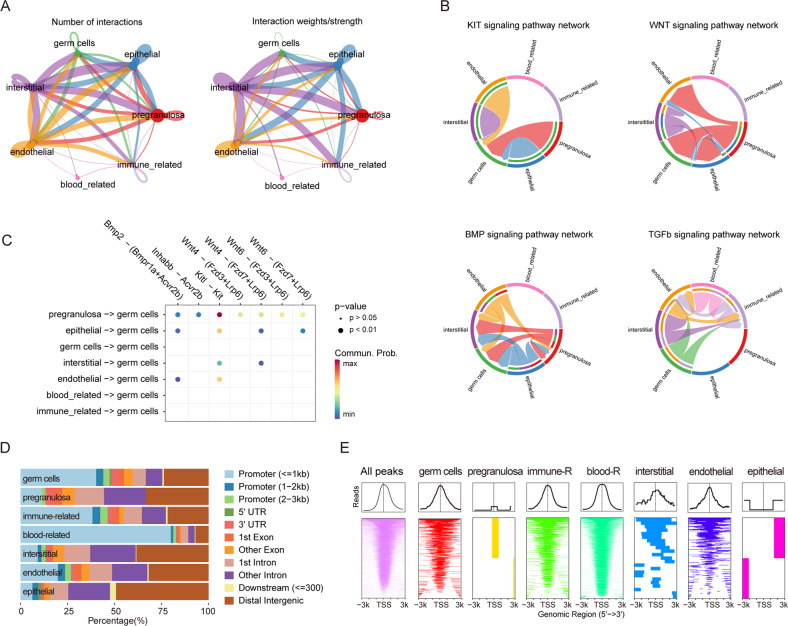
Transcription and chromatin accessibility characteristics of cells from E11.5–13.5 ovaries. **A** Cellchat of the ligand-receptor pairs across seven cell types during E11.5 - E13.5 embryonic ovary; Left shows the number of ligand-receptor pairs and the right shows the interaction strength. **B** Four important signaling pathways targeting germ cells, including KIT, WNT, BMP, and TGFβ signaling pathways; The width of the line shows the interaction strength. **C** The heatmap shows important ligand-receptor pairs for targeting germ cells; Red represents strong interactions and blue metabolizes weak interactions. **D** Location distribution of different cell type-specific peaks. **E** Relationship between transcription starting site and peaks and different cell type-specific peaks, from left to right are all detected peaks (pink), the specific peaks of germ cells (red), pregranulosa cells (orange), immune-related cells (chartreuse), blood-related cells (light green), interstitial cells (blue), endothelial cells (purple), and epithelial cells (magenta); TSS, transcription starting site.

Since E11.5–13.5 is a critical period for germ cell development characterized, among others, by a variety of interactions with the surrounding cells [36, 37], in order to validate our analyses, we analyzed the scRNA-seq data using CellChat for cell communication analysis. We found a strong ligand-receptor interrelationship between germ cells and interstitial cells (Fig. 2A). Namely, in line with previous studies [38, 39], we identified four main signaling pathways targeting germ cells, including KIT, WNT, BMP, and TGFβ (Fig. 2B). Significantly, we also evidenced a strong interaction between pregranulosa cells and germ cells through *Kitl-Kit* signaling (*p* < 0.01) (Fig. 2C). Finally, interactions of interstitial cells and epithelial cells with other cell types were also apparent (Supplementary Fig. S3A).

### Chromatin status and transcription dynamics of the ovarian cell populations

It is well established that chromatin opening at promoters is positively correlated with gene expression. In this regard, our scATAC data showed that between E11.5–13.5 in germ cells, about 50% of open chromatin regions are located at promoters (Fig. 2D). The heatmap of chromatin accessibility at transcription start sites confirming these results is shown in Fig. 2E. The specificity of the transcriptome of each cell type was confirmed by differentially expressed peak (DEP) analysis that showed unique characteristics in each different cell type (Supplementary Fig. S3B–G).

**Fig. 3:**
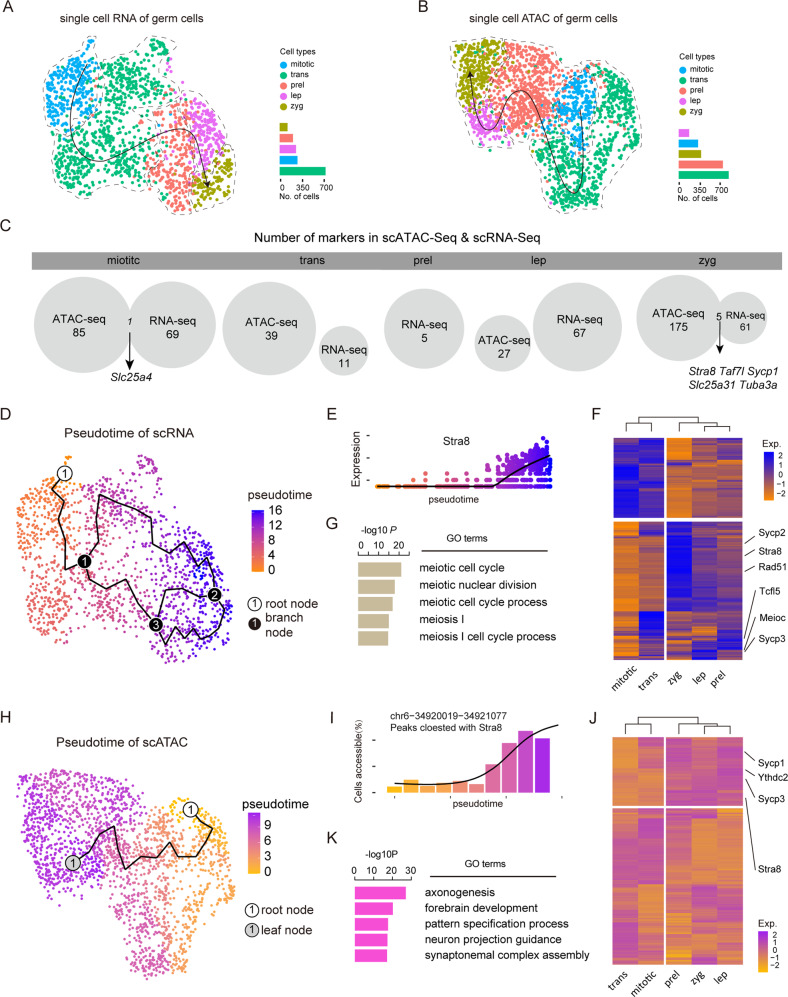
Identification of female germ cell subclusters. **A**, **B** Single-cell RNA (**A**) and single-cell ATAC (**B**) profiles of germ cells; The barplot on the right shows the number of cells in different cell subtypes; The solid black lines represent developmental paths and the arrows represent developmental directions; mitotic mitotic subtypes, trans transition-phase subtypes, prel preleptotene subtypes, lep leptotene subtypes, zyg zygotene subtype. **C** Number of markers in scATAC-seq and scRNA-seq in the five germ cell subtypes. **D** Pseudo-time of germ cells in the transcriptome, the root node indicates a development starting point and branch nodes indicate a development direction. **E** *Stra8* expression in pseudo-time. The solid black lines represent developmental paths, and the size of the number represents the order of occurrence. **F**, **G** The heatmap (**F**) of the genes in pseudo-time level and gene ontology annotation (**G**) of upregulation genes in pseudo-time, respectively; Right shows the upregulation genes. **H** Pseudo-time of germ cells in chromatin accessibility, the root node represents the starting point of development and the leaf node indicates the development of fate. **I** Expression of peak related to *Stra8* in pseudo-time. **J**, **K** The heatmap of the peaks in pseudo-time level and gene ontology annotation of upregulation peaks related genes in pseudo-time, respectively; Right shows the upregulation peaks related genes.

For a precise characterization of the genomic status of germ cells at the mitotic-meiotic shift, we needed to accurately identify germ cell clusters. Using scRNA data, 1798 germ cells were divided into nine subclusters and, on the basis of gene expression markers, they were annotated into five cell subtypes, including mitotic cells (279 cells mainly in E11.5 ovaries, low meiosis gene expression, and high pluripotent gene expression) [2, 40], transition-phase cells (722 cells most in E12.5 ovaries without *Stra8* expression) [5], preleptotene cells (257 cells in E12.5 ovaries with low *Stra8* expression) [24], leptotene cells (209 cells in E13.5 ovaries with high *Stra8* expression) [5], and zygotene cells (124 cells in E13.5 ovaries with high expression of *Stra8* and other meiotic genes such as *Ugt8a* and *Meioc*) [41] (Fig. 3A and Supplementary Fig. S4A–C). Moreover, transcriptome dynamics analysis by RNA velocity revealed that gene expression trends changed from mitosis states (mitotic, transition-phase) to meiosis states (preleptotene, leptotene, and zygotene) (Supplementary Fig. S4D). For scATAC data, 2298 cells were divided into nine subclusters, which were also annotated into five cell subtypes, including 282 mitotic cells, 727 transition-phase cells, 642 preleptotene cells, 151 leptotene cells, and 325 zygotene cells (Fig. 3B and Supplementary Fig. S4E, F).

We investigated the distribution of cell types at different time points and found preleptotene status in the scATAC data of E11.5 and in the scRNA data of E12.5, which supports variations in chromatin accessibility and gene expression (Supplementary Fig. S4G, H). We next analyzed differentially expressed genes (DEGs) and DEPs for all germ cell subtypes. The results showed that, in zygotene cells, there was little overlap between DEG and DEP genes, except for a few genes, including *Stra8* and *Slc25a31*, the latter is a mitochondrial translocator of the ADP/ATP carrier family that specifically expressed in male meiotic germ cells [42] (Fig. 3C).

Trajectories analysis of the germ cell transcriptome showed three branching nodes representing the direction of the developmental trajectory throughout development without leaf nodes that represent the fateful endpoints of development. Thus providing evidence that, despite a robust progressive transcriptional amplification effect, the mitotic to meiotic shift is a gradual rather than a sharp transition (Fig. 3D). In this regard, the *Stra8* expression level increased with that of pseudo-time, which is consistent with our previous observation (Fig. 3E). Heatmaps of key genes regulating meiotic initiation showed that the mitotic and transition phases were significantly different from the meiotic phases (preleptotene, leptotene, and zygotene) (Fig. 3F). Moreover, we observed highly variable gene expressions (i.e., for *Stra8*, *Sycp3*, *Meioc*, and *Tcfl5*) altering fate trajectories and that, as expected, were closely associated with meiotic processes (Fig. 3F, G). scATAC data were analyzed for the same trajectories and were consistent with scRNA-seq data, including the accessibility of the *Stra8* promoter region that increased with pseudo-time (Fig. 3H, I). The heatmap of key peaks regulating meiotic initiation also showed highly variable peaks (i.e., for *Stra8*, *Sycp3*, and *Ythdc2*) that, were involved in synaptonemal complex assembly (Fig. 3J, K).

### Chromatin status and transcriptome dynamics of germ cells throughout preleptotene and leptotene stages

**Fig. 4:**
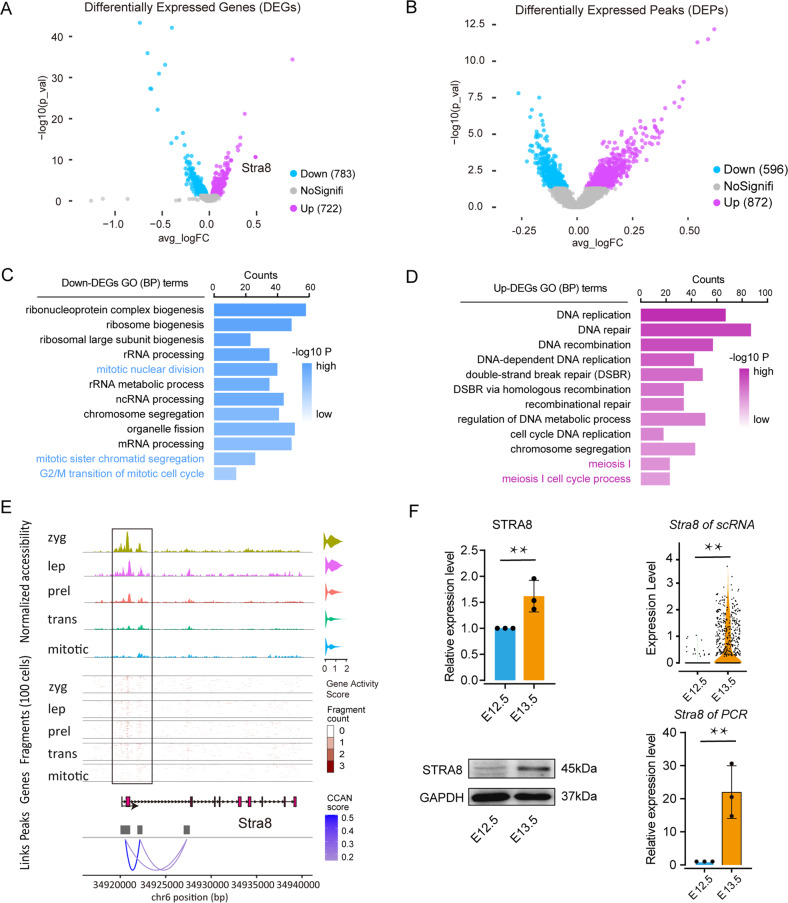
Transcriptome and chromatin accessibility of female germ cells during mitotic/meiotic switch. **A**, **B** Volcano plot of differentially expressed genes (DEGs) and differentially expressed peaks (DEPs): leptotene vs. preleptotene, respectively. Celeste represents downregulation and purple represents upregulation. **C**, **D**, GO annotation of down- and up-regulated DEGs, respectively. **E** Chromatin accessibility of peak related to *Stra8*; Volcano plot shows the gene activity score of *Stra8* in cell subtypes; From top to bottom, the epigenetic characteristics of *Stra8*, the density of the open region of the middle surface, the structure of the *Stra8* gene, the position of the peaks, and the cis-co-accessible networks of stra8 related peaks; mitotic mitotic subtypes, trans transition-phase subtypes, prel preleptotene subtypes, lep leptotene subtypes, zyg zygotene subtype. **F** Different levels of *Stra8* expression between E12.5 and E13.5, including transcript (PCR, *n* = 3 and scRNA) and protein (WB, *n* = 3); Compared to E12.5, ** means *p* value < 0.01.

Focusing on the preleptotene and leptotene stages, we observed dramatic changes in both the epigenome and transcriptome. Namely, the transcriptome had 1505 DEGs, including 722 up-DEGs and 783 down-DEGs, while the number of DEPs in the epigenome was 1468, with 872 up-DEPs and 596 down-DEPs (Fig. 4A, B, see detail in Supplementary Table S1). Coherently, among DEGs, we found down-DEGs related to the mitotic cell cycle, such as mitotic nuclear division (GO:0140014), while up-DEGs were associated with meiotic processes, such as meiosis I (GO:0007127) (Fig. 4C, D). About *Stra8*, we observed that the accessibility of its promoter region increased almost linearly during the transition from mitosis to meiosis while a dramatic increase of transcription and protein expression occurred between E12.5 and E13.5 (Fig. 4E, F).

### Identification of TFs network regulating mitotic/meiotic switch

**Fig. 5:**
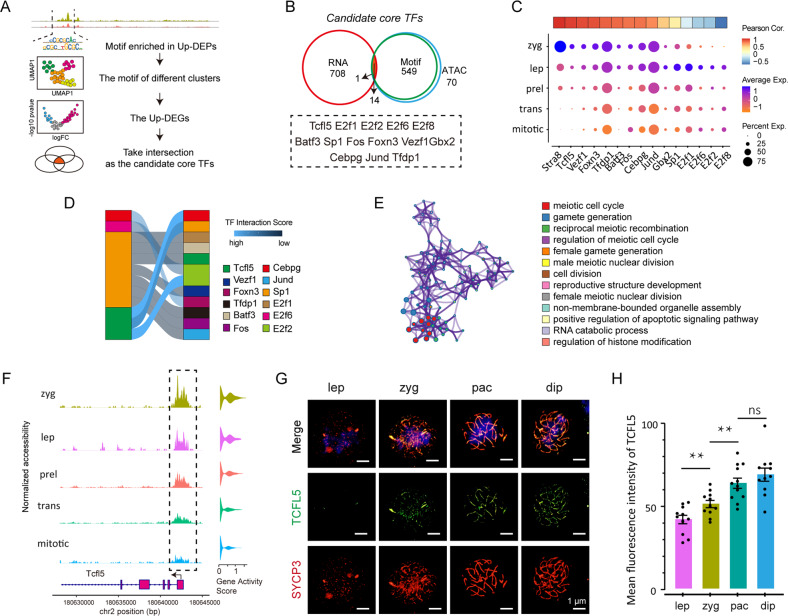
Identification of TFs in female germ cells associated with mitotic/meiotic switch. **A** Workflow of the selected framework of potential core TFs in meiotic initiation. **B** Final acquired core TFs of meiotic initiation. **C** Dot plot shows the expression trend of final acquired potential core TFs. The upper panel shows Pearson’s correlation between core TFs and *Stra8*. **D** Sankey diagram of TFs interaction network; Blue represents strong interactions, black represents weak interactions. **E** Function enrichment network of TCFL5 potential bound genes. **F** The epigenetic characteristics of *Tcfl5*; Volcano plot shows the gene activity score of *Stra8* in cell subtypes. **G** TCFL5 immunolocalization on chromosomes of germ cells at different stages of meiotic prophase I; bar = 1 μm, *n* ≥ 11; Red shows SYCP3, green showsTCFL5, and blue shows nuclei; **H** The barplot showing the expression level of TCFL5 protein at different stages of meiotic prophase I. ** means *p* value < 0.01. mitotic mitotic subtypes, trans transition-phase subtypes, prel preleptotene subtypes, lep leptotene subtypes, zyg zygotene subtype, pac pachytene subtype, dip diplotene subtype.

In order to identify the core TFs expressed during the mitotic/meiotic switch, we established a selection framework (Fig. 5A, see details in Materials and Methods). First, we scanned TF motifs in DEPs of preleptotene and leptotene cells and found that among the top ten motifs, that of TCFL5 showed higher fold enrichment (Supplementary Fig. S5A). Next, using chromVAR, we directly observed TF motif expression in all cell subtypes and found that some motifs were significantly increased at the leptotene stage, such as those of the DMRT family (Supplementary Fig. S5B). Then, we intersected DEPs, TF motifs, and DEGs to obtain core TFs. Using such a strict screening workflow, 14 candidate core TFs associated with mitotic/meiotic switch were obtained, including basic TFs such as SP1, E2F2, and TCFL5 (Fig. 5B). When the expression pattern of each of these TFs was reported as dot blots in the function of the developmental stages, surprisingly, we found that the expression pattern of *Tcfl5* was highly consistent with that of *Stra8* (Fig. 5C).

To reveal possible interactions among TFs, we next used a method termed TF interaction score (for details in Materials and Methods). This analysis revealed that TCFL5 was able to interact strongly with other core TFs, including E2F2, SP1, and CEBPG (Fig. 5D). Scanning at the genome-wide level the peaks containing TCFL5 motifs and GO analysis of the related genes indicated as potentially TCFL5-activable primarily genes involved in regulating the meiotic cell cycle (Fig. 5E). All TCFL5-potentially activable genes are shown in Supplementary Table S2. We next explored TCFL5 expression in terms of chromatin accessibility as reported in Fig. 5F. The results were compatible with low chromatin accessibility at mitosis and conversely progressively higher chromatin accessibility at meiotic stages (Fig. 5F). Moreover, immunolocalization of TCFL5 showed that its presence on chromosomes clearly increased from leptotene to diplotene (Fig. 5G, H). Finally, we explored the expression characteristics of TCFL5 during mitotic/meiotic switch from different aspects, including footprinting, transcriptic, motif distribution, and epigenetic. The results showed that once in meiosis, TCFL5 was highly activated (Supplementary Fig. S5C).

### *Tcfl5*^*+/−*^ mice are sterile

**Fig. 6:**
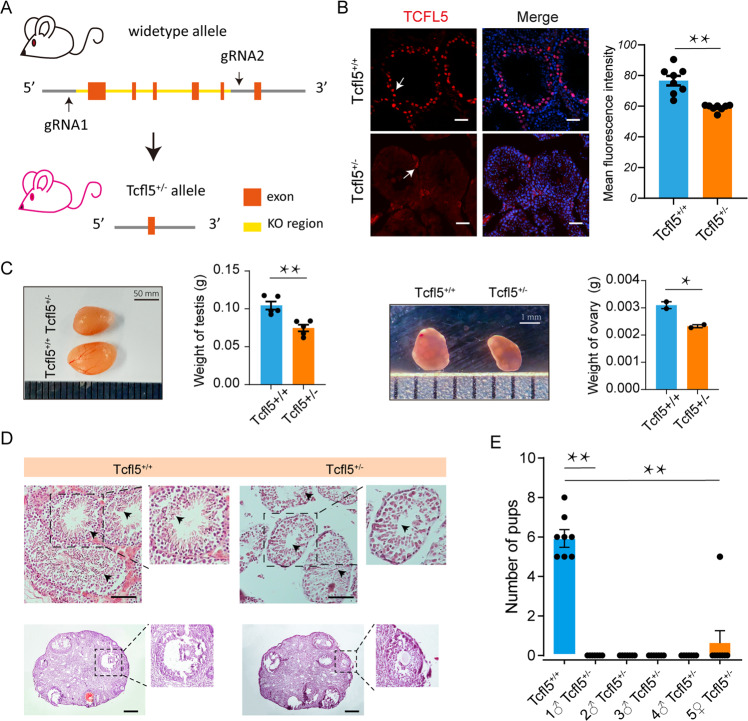
Characterization of *Tcfl5*^+/-^ mice. **A** Construction strategy of *Tcfl5*^+/−^ mice; Orange represents exons; The yellow area represents the knockout area. **B** TCFL5 immunolocalization of germ cells in *Tcfl5*^*+/−*^ male mice; bar = 50 μm, *n* ≥ 3; Compared to wild type, ** means *p* value < 0.01. **C** Morphology and weight of testis (left) and ovary (right) of *Tcfl5*^*+/−*^ mice; Compared to wild type, * means *p* value < 0.05 and ** means *p* value < 0.01. **D** Observation of testicular seminiferous tubules (up) and ovary (down) in *Tcfl5*^*+/−*^ mice and normal mice according to HE staining. bar = 50 μm. **E** Statistics of litter size of *Tcfl5*^*+/−*^ mice; Compared to wild type, ^**^ means *p* value < 0.01.

Based on the above results, we speculate that TCFL5 acts as a key TF during the mitotic/meiotic switch. To confirm our hypothesis, CRISPR/Cas9 gene editing technology was used to delete the exon 1–5 region of the *Tcfl5* gene and to establish a *Tcfl5*-heterozygote mouse model (Fig. 6A). Five *Tcfl5*^*+/*−^ mice were obtained, including four males and one female. Immunofluorescence staining of *Tcfl5*^*+/−*^ adult testes showed reduced staining in comparison to wild-type mice compatible with haploinsufficiency (Fig. 6B). Moreover, compared with wild-type mice, *Tcfl5*^*+/−*^ mice have significantly smaller testes and ovaries (Fig. 6C). Histomorphological analysis revealed that fewer sperm were present within seminiferous tubules of Tcfl5^+/−^ males compared to wild-type (Fig. 6D). Conversely, no obvious difference was observed in follicle numbers and classes between wild-type and Tcfl5^+/−^ adult females (Fig. 6D). However, both Tcfl5^+/−^ males and females showed impaired fertility. Namely, during one year of repeated mating, Tcfl5^+/−^ males were sterile while Tcfl5^+/−^ females generated a greatly reduced number of pups (Fig. 6E).

### Silencing *Tcfl5* expression by RNA interference delays meiotic prophase I progression in embryonic ovaries in vitro

**Fig. 7:**
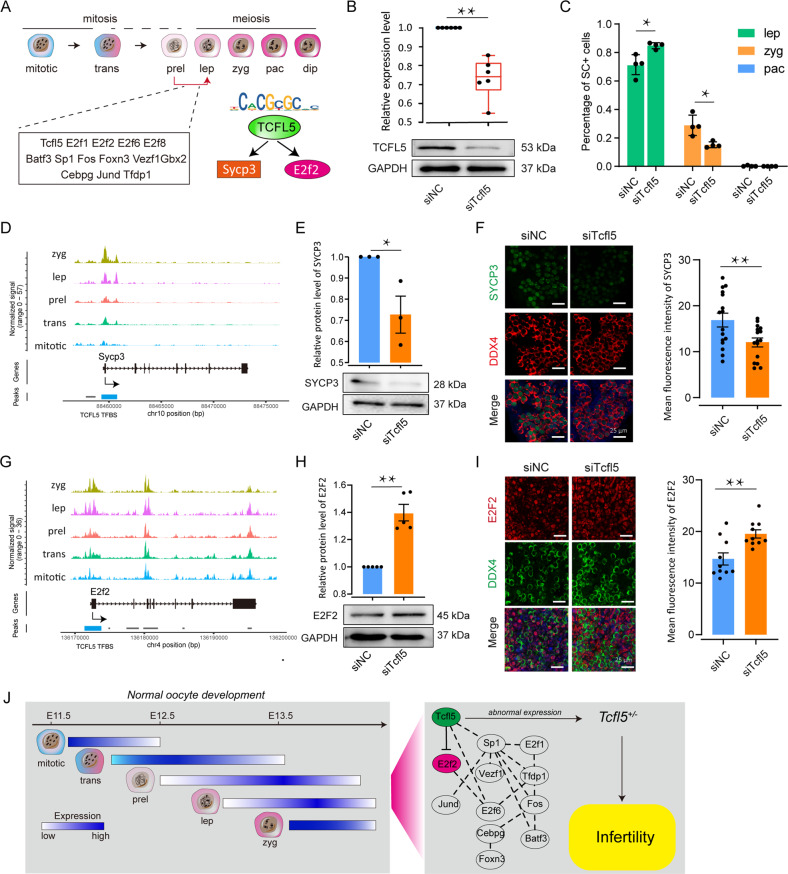
The potential mechanism of TCFL5 in mitotic/meiotic switch. **A** The model shows the potential downstream factors regulated by TCFL5 in the mitotic/meiotic switch. **B** Effect of *siTcfl5* on TCLF5 expression; *n* ≥ 3; Compared to siNC group, ** means *p* value < 0.01. **C** Effect of *siTcfl5* to meiotic progression on in vitro cultured E12.5 ovaries; *n* ≥ 3; * means *p* value < 0.05. **D** the epigenetic characteristics of *Sycp3*, and the TCFL5 binding site (blue bar) was found in the promoter region of *Sycp3*. **E**, **F** The western blot and immunofluorescence staining indicated the expression level of SYCP3 after *siTcfl5* on in vitro cultured E12.5 ovaries; n ≥ 3; Compared to siNC group, * means *p* value < 0.05 and ** means *p* value < 0.01. **G** The epigenetic characteristics of *E2f2*, and the promoter region with TCFL5 binding site (blue bar). **H**, **I** the western blot and immunofluorescence staining indicated the expression level of E2F2 after *siTcfl5* on in vitro cultured E12.5 ovaries; *n* ≥ 3; Compared to siNC group, ** means *p* value < 0.01. **J** Molecular events and underlying TF regulatory networks during mitotic/meiotic switch, represented by TCFL5. The left panel shows that the female germ cell mitotic/meiotic switch is a transcriptional amplification event; the middle panel shows the transcription factor regulatory network in this event; the right panel shows the transcription factor regulatory mechanism represented by TCFL5.

Since the bioinformatics data suggested that TCFL5 interacts with the *E2f2* and regulates *Sycp3* expression during the first stages of meiotic prophase I (Fig. 7A), we investigated the effect of silencing TCFL5 expression with RNA interference on this meiotic phase in in vitro cultured ovaries and aimed to identify possible binding sites for TCFL5 on *E2f2* and *Sycp3* promoters. The results showed that when E12.5 ovaries were transfected and cultured for 24 h with *siTcfl5*, the expression of TCFL5 was significantly reduced (Fig. 7B) and the meiotic progression of oocytes from leptotene to zygotene was significantly delayed (Fig. 7C). Next, we identified TCFL5 binding sites in the promoter region of *Sycp3* (Fig. 7D) and found that the expression of SYCP3 was significantly reduced when ovaries were transfected with *siTcfl5* (Fig. 7E, F). As expected, TCFL5 binding sites were present in the promoter region of *E2f2* (Fig. 7G), however, contrarily to SYCP3, *siTcfl5* increased E2F2 protein expression (Fig. 7H, I).

### Meiotic entry primarily involves the amplification of a distinctive germ cell transcription program

On the basis of Kojima and coll. reports showing that in the mouse germ cells of the adult testes, the mitotic/meiotic switch is driven by the amplification of extensive transcriptional programs rather than the expression of new genes [5], we hypothesized a similar event in the embryonic ovaries. To address this question, we reanalyzed available published data from mouse embryonic ovaries [24] and found that, with only a few exceptions, for example, *Stra8*, the results supported such a notion (Supplementary Fig. S6A–D). Considering the average expression of genes in germ cells from mitotic (E11.5), transition (E12.5), and meiotic (preleptotene and leptotene; E13.5) stages, it is possible to calculate that about 85% of the genes upregulated at leptotene were expressed at significant levels before meiotic initiation (Supplementary Fig. S6D). Notably, although the expression of *Stra8* was not detectable before preleptotene, that of other key meiotic genes was already evident at the mitotic or transition (i.e., *Sycp3*) stages and dramatically increased thereafter. Moreover, we observed that genes such as *Stra8*, *Tcfl5*, *Sycp3*, and *E2f5* showed an open chromatin status already at the mitotic stage (Supplementary Fig. S6E, F).

## Discussion

Recent advances in single-cell technologies have enabled the measurement of gene expression and chromatin accessibility at a single-cell resolution using scRNA-seq and scATAC-seq. They are providing unprecedented opportunities to investigate the intricate gene regulation mechanisms underlying complex cellular processes such as the initiation and progression of meiosis in germ cells [2, 43]. Regulation of meiotic initiation and progression throughout prophase I during germ cell development remains poorly defined in mammals, particularly in females in which these processes occur within less accessible embryonic ovaries.

Here we applied scRNA-seq and scATAC-seq to over 18,000 single cells obtained from female gonads of E11.5–13.5 mouse embryos. According to the expression of marker genes, seven distinct cell types, namely germ cells, pregranulosa cells, epithelial, interstitial cells, endothelial cells, blood-related cells, and immune-related cells, were identified [2]. Interestingly, searching scRNA-seq with CellChat for the ligand-receptor communication algorithm, reinforced and enlarged the notion of a complex network of interactions among the majority of the ovarian cell populations, primarily between somatic cells and germ cells, including KIT, WNT, BMP, and TGFβ signaling pathways, during the critical period of ovarian development centered on the beginning of meiosis. Focusing on germ cells, both for scRNA-seq and scATAC-seq, we identified nine subclusters that were annotated into five cell subtypes including mitotic, transition-phase, preleptotene, leptotene, and zygotene cells. We observed that, as expected, a large majority of DEGs and DEPs of all germ cell subtypes showed little overlap supporting the notion that chromatin accessibility represents only one level of transcriptional regulation [44]. The trajectory of the germ cell transcriptome during development was compatible with a gradual transition from a mitotic to a meiotic cycle associated with robust progressive transcriptional amplification [5]. The latter was mainly related to meiotic cell cycle processes, such as increased expression of *Stra8* [5].

The fact that TFs are important hubs to regulate cell fate transition has been well established [45]. Adopting a strict TF screening framework that makes it easier to understand the single-cell chromatin signature and a TF interaction algorithm that integrates the transcript levels, chromatin accessibility, and motif scores, we identified 14 TFs potentially regulating the mitotic/meiotic switch, including TCFL5, E2F1, E2F2, E2F6, E2F8, BATF3, SP1, FOS, FOXN3, VEZF1, GBX2, CEBPG, JUND, and TFDP1. Among these, the basic helix-loop-helix motif transcription factor TCFL5 was already known to play a master role in male germ cell meiosis [46, 47]. Our results suggested a regulatory role of TCFL5 also in meiotic prophase I of oocytes. First, the TCFL5 motifs showed the highest fold enrichment among the top ten TF motifs in DEPs of preleptotene and leptotene cells. Second, only TCFL5 was able to strongly interact with other core TFs. Third, the functional enrichment analysis of peaks containing TCFL5 motifs revealed terms related to the meiotic cell cycle. Based on these indications, we constructed *Tcfl5*^*+/−*^ mice using the CRISPR/Cas9 technology. In line with two recent reports [47, 48], we found that male *Tcfl5*^*+/−*^ were sterile while *Tcfl5*^*+/−*^ females showed severe fertility impairment. Since we were unable to obtain embryonic ovaries from *Tcfl5*^*+/−*^ females, we employed in vitro cultured E12.5 ovaries form wild-type embryos to explore the underlying molecular mechanism of TCFL5 function at this stage. RNA interference experiments showed that *siTcfl5* increased the expression of E2F2 and inhibited the expression of SYCP3 in the cultured ovaries. Moreover, in line with this latter result, meiotic progression from leptotene to zygotene was significantly delated. As noted, binding sites for TCFL5 both on *E2f2* and *Sycp3* promoters were identified. The report that in male testis TCFL5 deficiency increased expression of meiotic genes (*Syce1, Stag3, and Morc2a*) and that male germ cells assembled apparently normal synaptonemal complexes but are unable to progress from pachytene to diplotene [48], suggest a different function of TCFL5 in the meiotic prophase I of oogenesis and spermatogenesis. The fact that in females, TCFL5 deficiency in the in vitro cultured ovaries affects the progression from leptotene to zygotene rather than the beginning of meiosis while *Tcfl5*^*+/−*^ adult females, despite apparently normal follicle classes in the ovary, are almost infertile, suggest poor oocyte quality. Further studies are needed to clarify this important point.

Since, Kojima and coll. reported that in male mice germ cells amplification of a broad transcriptional program by STRA8 rather than the expression of new genes triggers the initiation of meiosis [5], we hypothesized a similar event in female meiosis independently orchestrated first by STRA8 (preleptotene-leptotene) and subsequently by TCFL5. (leptotene-zygotene) In this regard, reanalyzing published datasets from mouse female ovaries [21], and our results, we found that actually more than 85% of the genes upregulated at the leptotene stage were expressed in germ cells at significant levels before meiotic initiation and that genes such as *Stra8*, *Tcfl5*, *Sycp3*, and *E2f5* showed an open chromatin status already at the mitotic stage.

In conclusion, 14 potential TFs regulating the first stages of the meiotic cell cycle in female germ cells entering into meiosis have been identified (Fig. 7J). The role played by one of them, namely TCFL5, probably in amplifying a broad meiotic transcriptional program in the transition from leptotene to zygotene, was empathized. This information, together with the identification of gene expression and chromatin status changes at single-cell resolution during these critical oogenesis stages, will hopefully provide new targets for understanding ovary pathologies in women. Among these, POI, a complex multifactorial ovary disorder involving multiple gene variants [12] and leading to the reduction of the primordial follicle pool [49]. In fact, more than 80 causative genes have been identified in POI, some of which are involved in the regulation of meiosis [12, 14].

## Materials and methods

### Animals

All animal experiments were approved by the Ethics Committee of Qingdao Agricultural University. All C57BL/6 mice were housed in an acclimatized environment with free access to food and water, and female fetuses were dissected at E11.5–13.5 for the isolation of their gonads. *Tcfl5*^+/−^ mouse model purchased from Cyagen Co., Ltd. (S-KO-08938). Briefly, CRISPR/Cas9 technology was used to delete exons 1–5 of the *Tcfl5* gene. Notably, the presence of a vaginal plug was recorded as E0.5 after mating and all experimental samples contained no mesonephric tissue.

### In vitro culture of gonads and siRNA

Isolated E12.5 ovaries, without mesonephric tissue, were cultured in vitro in a humidified incubator at 37 °C and 5% CO_2_ in the air, in MEM alpha medium (HyClone, SH30265.01, China) supplemented with 10% FBS (Gibco, 10099-141, Australia), 1% penicillin-streptomycin (HyClone, SV30010), 1% sodium pyruvate (HyClone, SH30239.01, China), and 0.24 IU/μl FSH (R&D Systems, 5925-FS-010, USA). Ovaries were individually cultured in 0.5 ml of medium in 24-tissue culture wells (NEST, 702001, China) for 48 h.

The siRNA sequence of the *Tcfl5* gene was generated by Shanghai GenePharma Co., Ltd. Ovaries were transfected with 20 μM si*Tcfl5* or the negative control for 24 h according to the manufacturer’s protocols. The interference efficiency was verified by Western blot. The si*Tcfl5* sequences were: (*Forward*) 5′-GCAGAGUUCUAGUAACUCATT-3′ and (Reverse) 5′-UGAGUUACUAGAACUCUGCTT-3′, and the negative control was (Forward) 5’-UUCUCCGAACGUGUCACGUTT-3′ and (Reverse) 5′-ACGUGACACGUUCGGAGAATT-3′.

### Western blot

Proteins were extracted from gonads with RIPA lysis buffer (Beyotime, P0013C, China) and then boiled for 5 min as previously described [50, 51]. Primary antibodies, including rabbit anti-TCFL5 (1:400, Abcam, ab188075, USA), mouse anti-SYCP3 (1:1000, Abcam, ab97672, USA), rabbit anti-STRA8 (1:1000, Abcam, ab49405, USA), and rabbit anti-GAPDH (1:1000, Affinity, AF7201, China), were used. Secondary antibodies were HRP-conjugated goat anti-rabbit (1:1000, Beyotime, A0258, China) or mouse IgG (1:1000, Beyotime, A0216, China).

### Staining of meiotic I prophase chromosome spreads

To determine the stages of meiotic prophase I, immunofluorescence staining was performed for SYCP3 (anti-SYCP3, 1:200, Abcam, ab97672), using the previously described protocols [2]. Briefly, the female genital ridges were dealt with hypotonic solution (30 mM Tris, 50 mM sucrose, 17 mM citric acid, 5 mM EDTA, 2.5 mM dl-dithiothreitol, and 1 mM phenylmethanesulfonyl fluoride in water), and then they were incubated with PBS supplemented with 1% goat serum and 0.05 M Tris-HCl. Finally, they adopted immunofluorescence staining. Meiotic stages were classified as follows: sporadic SYCP3 signals (leptotene), fragmentary SYCP3 signals (zygotene), rough rod SYCP3 signals (pachytene), and rough rod with bifurcated SYCP3 signals (diplotene) [52–54]. Moreover, TCFL5 (rabbit anti-TCFL5, 1:200, Affinity, AF9211) expression levels during meiotic prophase I were also detected by ImageJ software (v1.51j8).

### HE and Immunofluorescence staining

Briefly, the testis was fixed with 4% paraformaldehyde, followed by wax dipping, dehydration, and fluorescent staining (anti-STRA8, 1:200, Abcam, ab49602; anti-TRA98, 1:200, Abcam, ab82527; anti-SYCP3, 1:200, Abcam, ab97672; anti-E2F2, 1:200, Affinity, AF4100). Immunofluorescence and HE staining steps were as previously described [50, 51].

### RNA extraction and qRT-PCR

Total RNA was extracted from gonads using a SPARKeasyTissue/Cell RNA kit (Shandong Sparkjade Biotechnology Co., Ltd., AC0201, China). RNA was then reverse transcribed into cDNA using a SPARKScript II RT Plus Kit (Shandong Sparkjade Biotechnology Co., Ltd., AG0304, China). The ABI 7500 Sequence Detection System was adopted for qRT-PCR, and the amplification conditions were as follows: 40 cycles with denaturing at 95 °C for 30 s, 95 °C for 5 s, and annealing at 60 °C for 34 s. The 2^−△△Ct^ method was used to calculate relative gene expression versus housekeeping *Gapdh*. The *Stra8* sequence was (Forward) 5′-ACCCTGGTAGGGCTCTTCAA-3′ and (Reverse) 5′-GACCTCCTCTAAGCTGTTGGG-3′. The *Gapdh* sequence was (Forward) 5′-AGGTCGGTGTGAACGGATTTG-3′ and (Reverse) 5′- TGTAGACCATGTAGTTGAGGTCA-3′.

### Construction of core TFs screening framework

TFs exert a key role in development, especially in determining cell fate [55], we adopt a rigorous screening framework to search for those TFs that are critical in regulating mitotic/meiotic switch. Firstly, we observed the motifs enriched in DEPs during the preleptotene turning to the leptotene stage by using Signac (v1.8.0) [56]. Next chromVAR directly scanned motifs in different stages, motifs that upregulated during leptotene vs. preleptotene were focused on scanning [57], and then the upregulation DEGs during the preleptotene turning to leptotene stage were obtained [58]. Finally, the intersections of DEPs, TF motifs, and DEGs were generated to obtain the core regulatory TFs.

### Construction of regulatory networks of TF interactions

Once the core regulatory TFs were available, we built a network of possible interactions among them. Assuming that TF1 regulated TF2, there was a TF1 motif at the TF2 promoter [59]. We stipulated that the TF1-TF2 interaction score = mean Exp. TF1 × mean motif score TF1 × mean TF2 peaks (Tn5 score). The Seurat *AverageExpression* function was used to calculate the mean score in different stages during the transition from mitosis to meiosis. A Sankey diagram was used to visualize the interaction of TFs. The ChIPSeeker *annotatePeak* function was adopted to locate the genes regulated by TFs, which had TF binding sites in the promoter region (transcription starting site ± 3 kb) [60].

### Statistical analysis

The data were exhibited as mean ± SEM and at least three biological replicates were performed. Differences were compared using unpaired t-tests and were conducted in GraphPad prism 8.

Bioinformatics analysis protocols are detailed in Supplementary Experimental Procedures.

## Supplementary information

checklist

FigS1

FigS2

FigS3

FigS4

FigS5

FigS6

Supplementary Experimental Procedures

Supplementary Table S1

Supplementary Table S2

Original Data File

## Data Availability

All data needed to evaluate the conclusions in the paper are present in the paper and/or the Supplementary Materials. All scRNA-seq data were derived from the GEO database, one of which was previously published by our laboratory (GSE128553) and the other was tested (GSE136441). The scATAC-seq raw data has been deposited in The National Genomics Data Center (NGDC) of the China National Center for Bioinformation (CNCB) and accession number: CRA004454.
