# Supplementary figures and images for: Single cell epigenomic and transcriptomic analysis uncovers potential transcription factors regulating mitotic/meiotic switch
Source: Cell Death Dis. 2023 Feb 17;14(2):134. doi: 10.1038/s41419-023-05671-w (PMC9935506; doi:10.1038/s41419-023-05671-w)

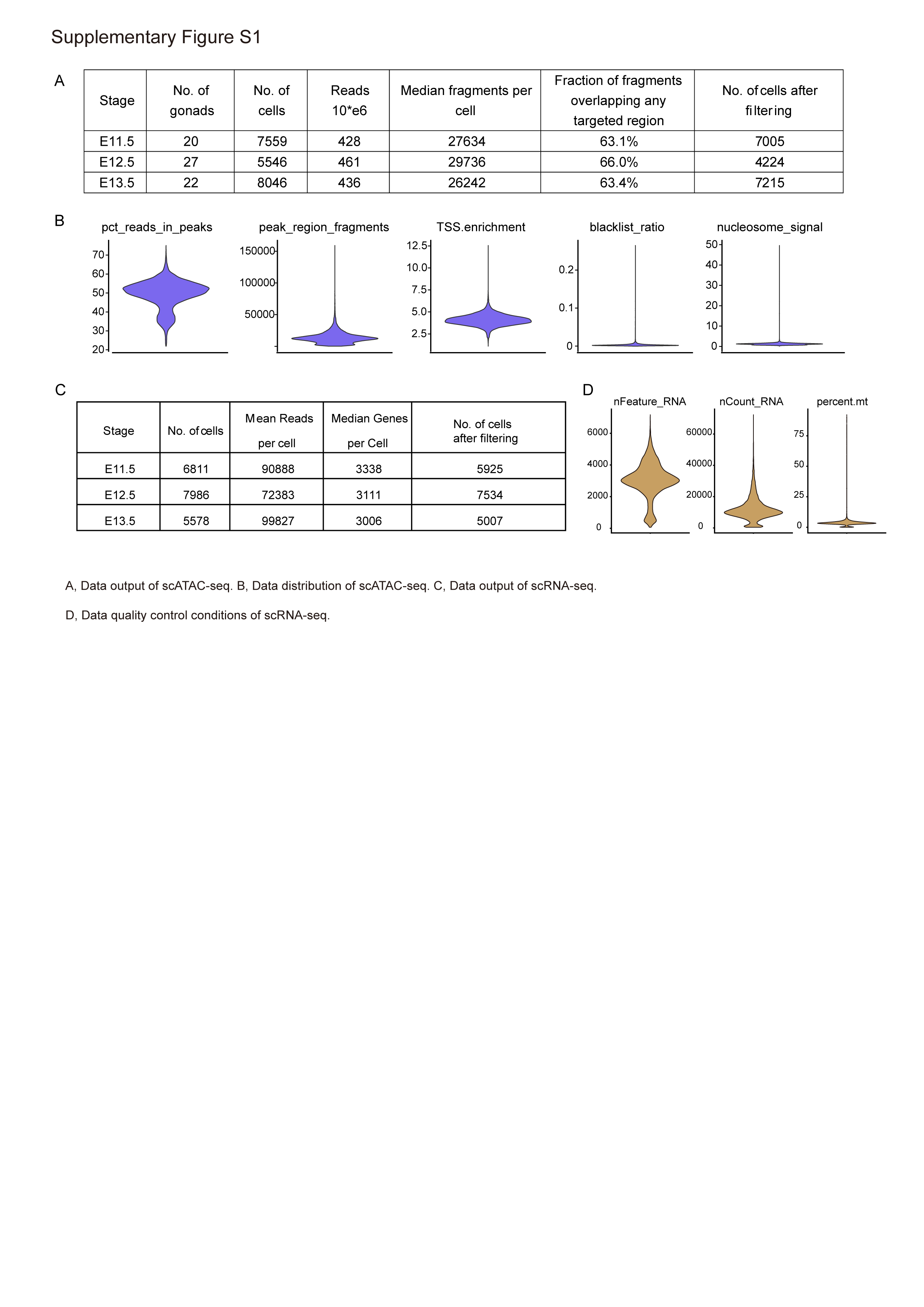

Supplement: Supplementary file 2 — FigS1 [file 41419_2023_5671_MOESM2_ESM.tif]

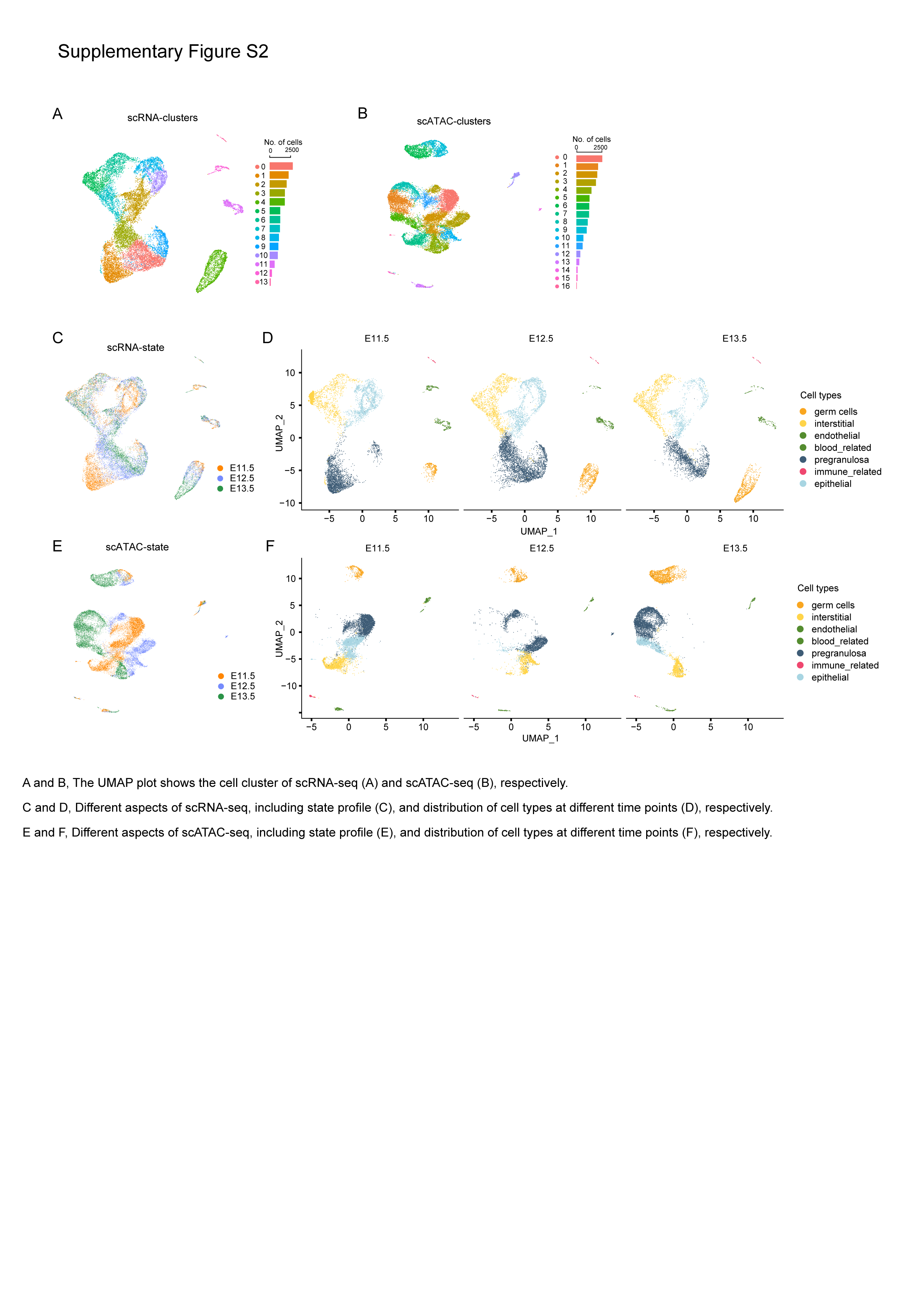

Supplement: Supplementary file 3 — FigS2 [file 41419_2023_5671_MOESM3_ESM.tif]

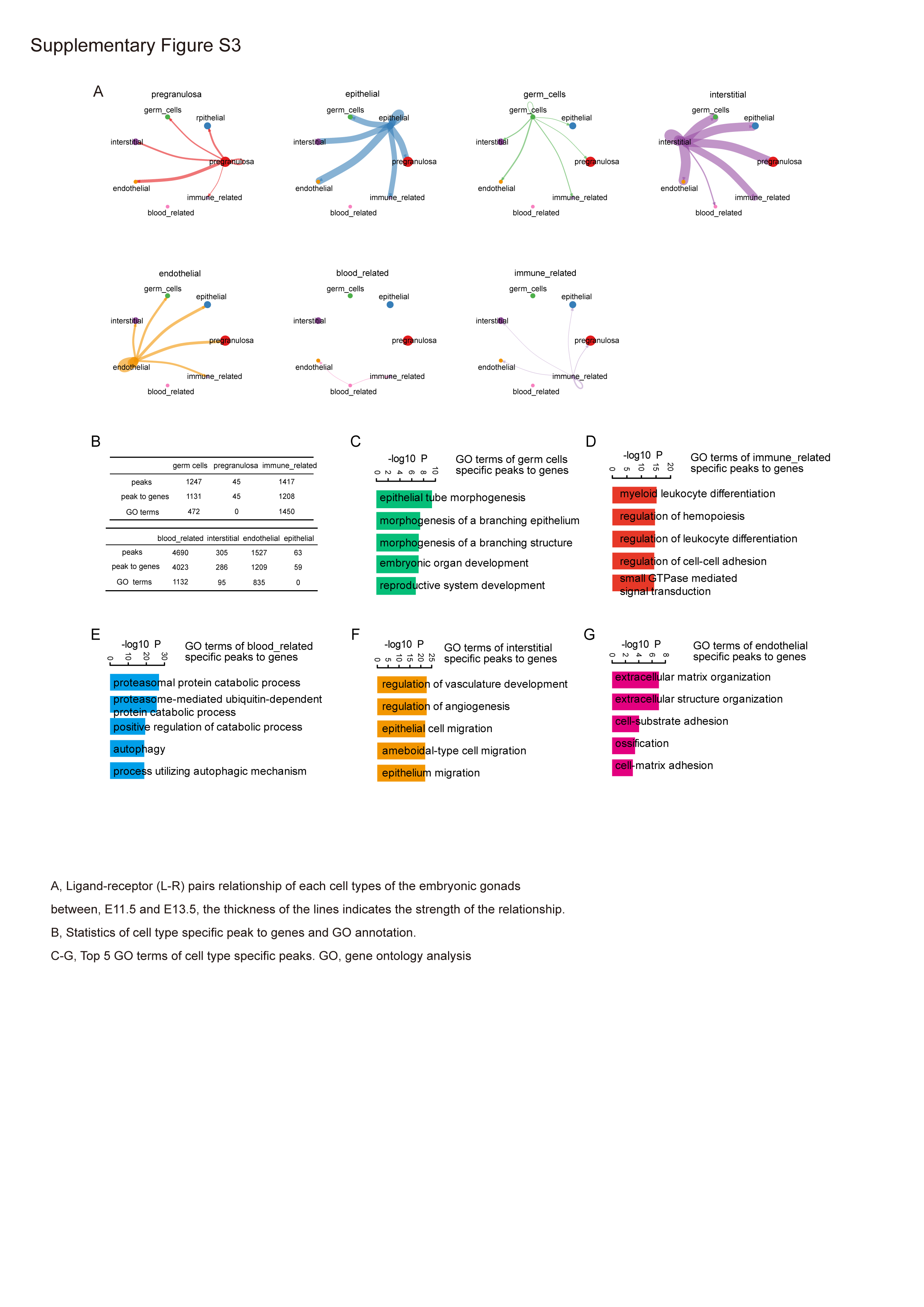

Supplement: Supplementary file 4 — FigS3 [file 41419_2023_5671_MOESM4_ESM.tif]

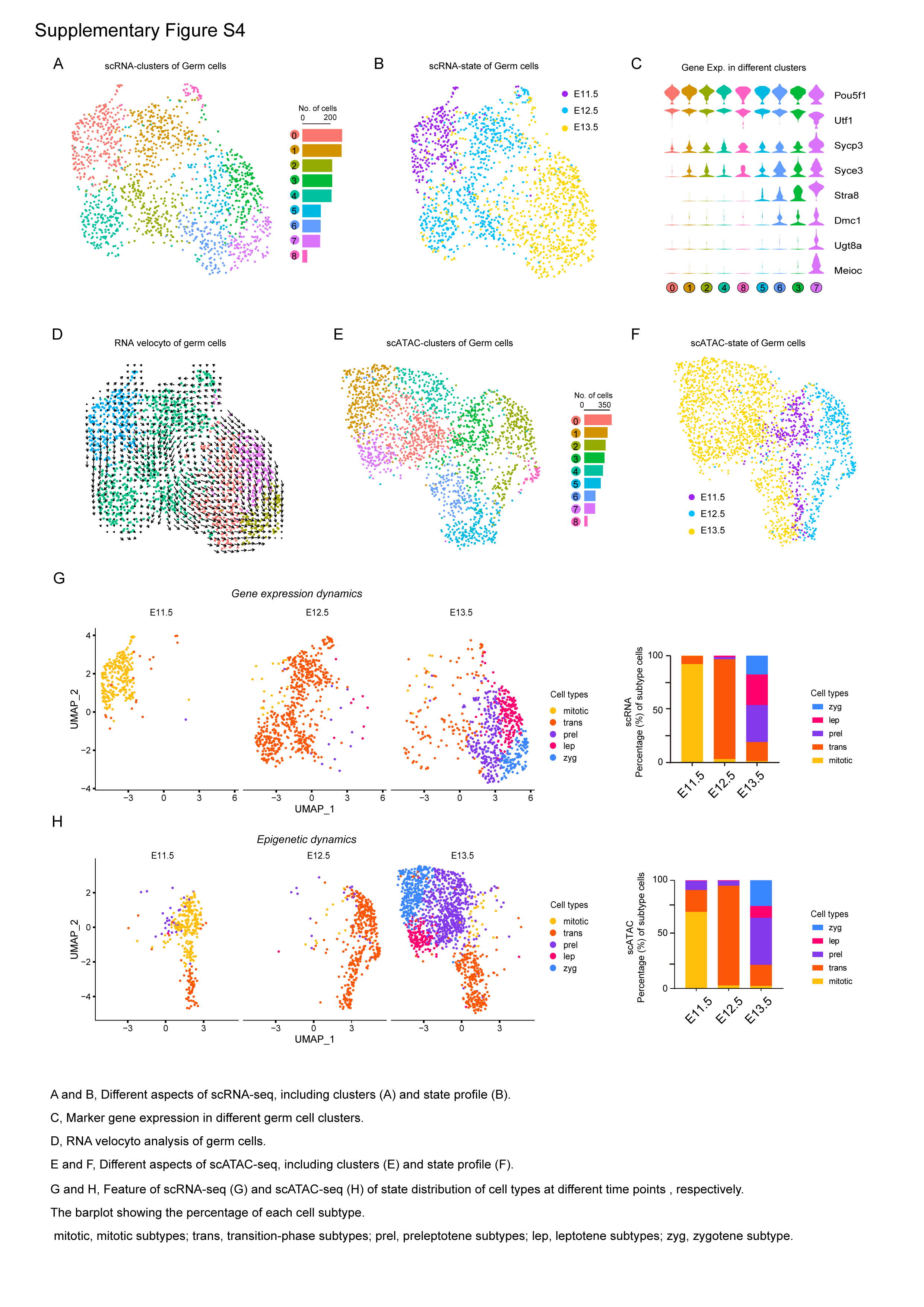

Supplement: Supplementary file 5 — FigS4 [file 41419_2023_5671_MOESM5_ESM.tif]

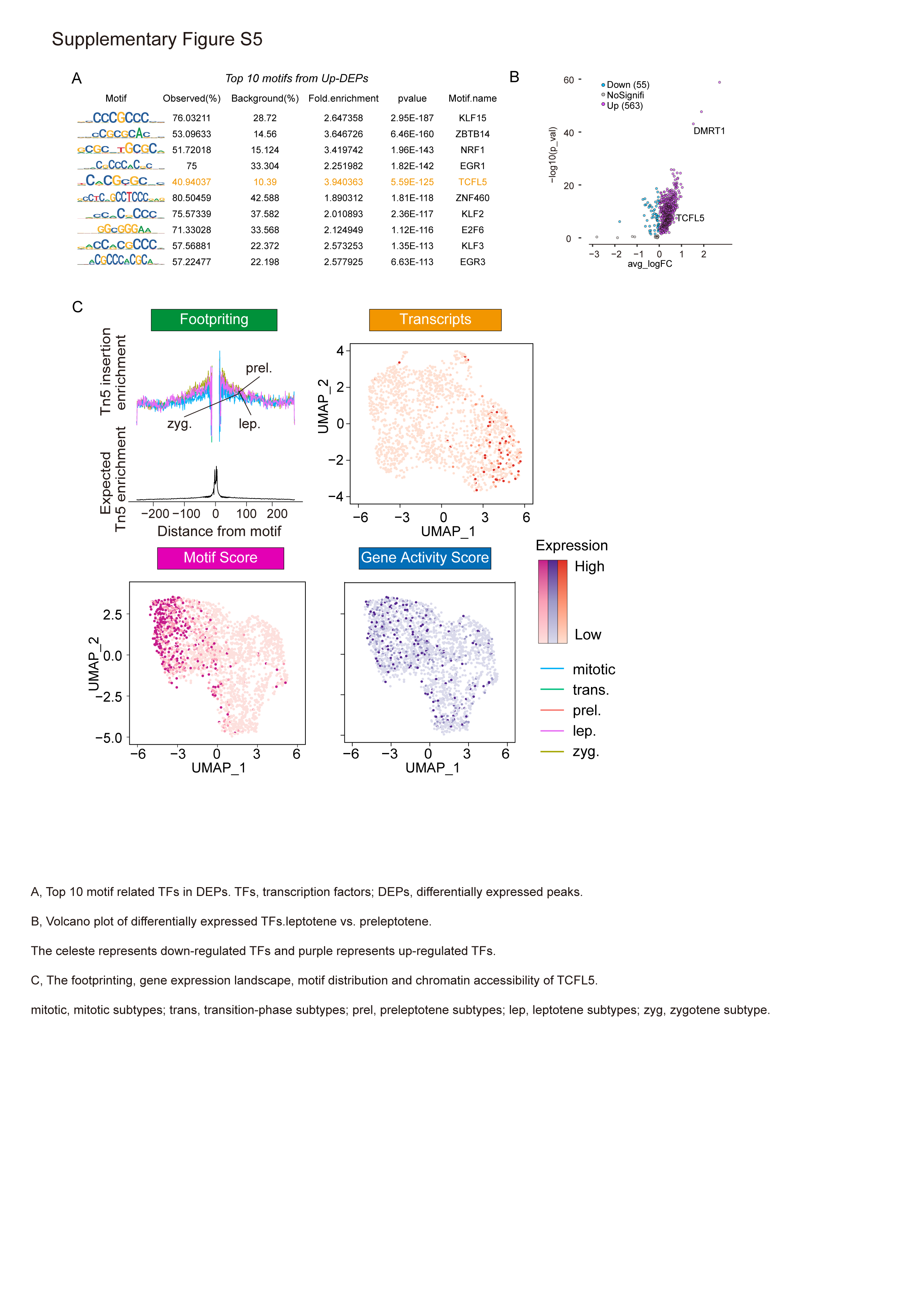

Supplement: Supplementary file 6 — FigS5 [file 41419_2023_5671_MOESM6_ESM.tif]

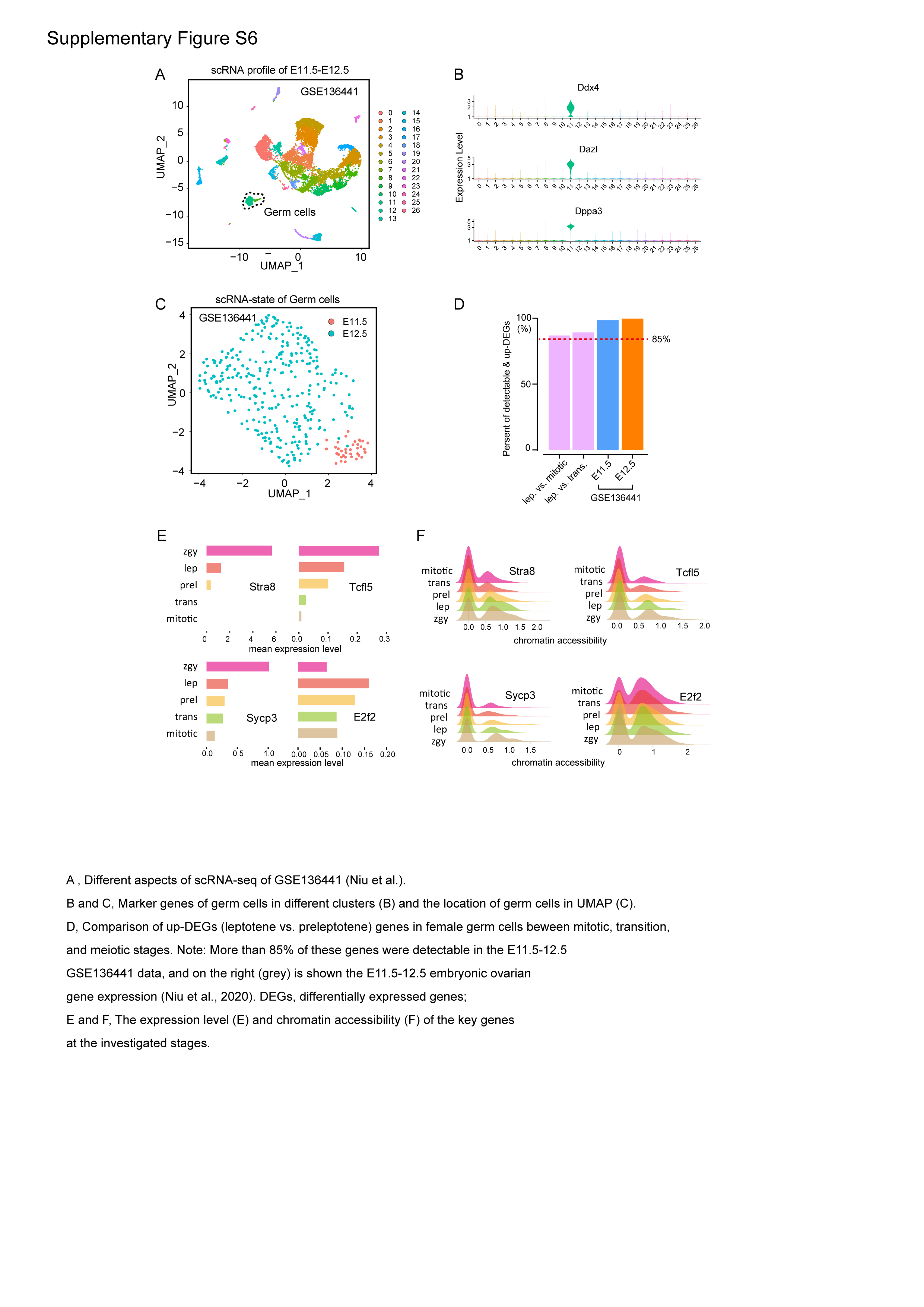

Supplement: Supplementary file 7 — FigS6 [file 41419_2023_5671_MOESM7_ESM.tif]

Fig 4F


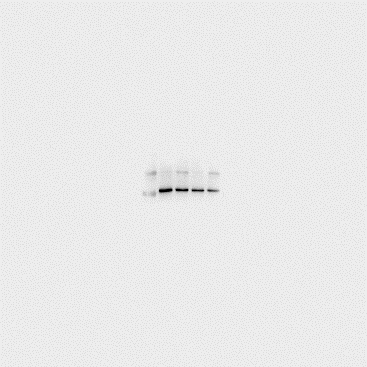


Fig 7B


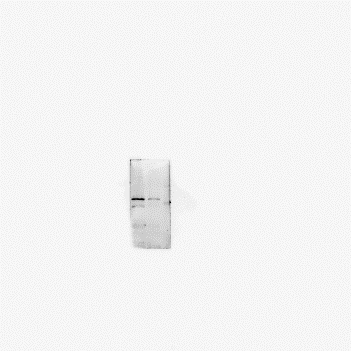

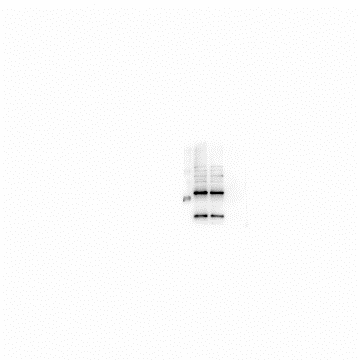


Fig 7E





Fig 7H


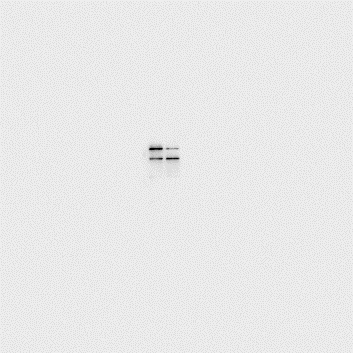

Supplement: Supplementary file 11 — Original Data File [file 41419_2023_5671_MOESM11_ESM.docx]
