# Supplementary material for: Single cell epigenomic and transcriptomic analysis uncovers potential transcription factors regulating mitotic/meiotic switch
Source: Cell Death Dis. 2023 Feb 17;14(2):134. doi: 10.1038/s41419-023-05671-w (PMC9935506; doi:10.1038/s41419-023-05671-w)
Supplement: Supplementary file 8 — Supplementary Experimental Procedures [file 41419_2023_5671_MOESM8_ESM.docx]

**Supplementary Experimental Procedures**

**scATAC-seq library preparation**

Assays for transposase-accessible chromatin using sequencing (scATAC-seq) libraries were prepared according to the 10× Chromium's library preparation scheme from single-cell suspensions isolated from gonads as previously described ^1^. At E11.5 the gonadal sex was determined using RT-PCR for *Sr*y and *Gapdh* as the housekeeping control: *Sry*: (*Forward*) 5′-CTG TGT AGG ATC TTC AAT CTC T-3′ and (*Reverse)* 5′-GTG GTG AGA GGC ACA AGT TGG C-3′ ^1, 2^; *Gapdh*: (*Forward*) 5′-AGG TCG GTG TGA ACG GAT TTG-3′ and (*Reverse)* 5′- TGT AGA CCA TGT AGT TGA GGT CA-3′.

Nuclei were isolated from the gonadal cells using 10× Genomics PN2000153 lysis buffer, and then scATAC-seq libraries were generated by using a chromium single cell ATAC V1 Gel Beads Kit (PN-1000111, 10× Genomics, USA).

**scATAC-seq bioinformatics workflow**

Three scATAC-seq libraries were sequenced using Illumina Novaseq apparatus. The raw data was analyzed with cellranger-atac *count* (10× Genomics, v1.2) using the mouse-mm10 reference genome to filter low quality data. Furthermore, cellranger-atac *aggr* was adapted to depth normalization in order to remove potential batch effects. Signac (https://github.com/timoast/signac, v1.1.0) and Seurat (v3.2.0), were used to process subsequent data ^3, 4^. The data filtering condition was *peak region fragments > 3000, peak region fragments < 40000, pct_reads in peaks > 30, blacklist ratio < 0.01, nucleosome signal < 4, and TSS.enrichment > 2.5*.

The vignettes detailed each class defined in Signac as reported in https://satijalab.org/signac/articles/mouse_brain_vignette.html. Briefly, the raw data was first quality-controlled and normalized; next, dimensionality reduction was carried out for the data, and then clustering with *resolution = 0.6* for all data and *resolution = 1* for germ cell data. Finally, uniform manifold approximation and projection (UMAP) was used for visualization. Genomic regions of scATAC-seq peaks were visualized by ChIPSeeker. The Seurat *FindMarker* function was used for difference analysis.

**scRNA-seq bioinformatics workflow**

Raw data were analyzed with cellranger *count* (10× Genomics, v3.1.0), using the mouse-mm10 reference genome to filter low quality data. Furthermore, cellranger *aggr* was adapted to depth normalization in order to remove potential batch effects. Seurat (v3.2.0) was used to process subsequent data ^3^. The data filtering condition was *nFeature_RNA > 1000 & nFeature_RNA < 6000 & percent.mt < 8.* Next, the data was dimensionalized, clustered with *resolution = 0.6* for all data and *resolution = 1* for germ cell data, and presented using UMAP.

**scATAC-seq cluster annotation**

To improve cluster annotation of scATAC-seq, firstly the Signac *GeneActivity* function was adopted to create gene activity matrixes, and then we used the Seurat *FindTransferanchors* function to match the scRNA-seq of the same biological samples to obtain a preliminary cluster annotation. Further, we observed chromatin accessibility at the promoter region of the marker gene of each cluster, the distribution of clusters at real developmental time, and the cluster distribution of pseudo-time to determine whether the annotation of the cluster was reasonable; it was then processed based on the comprehensive situation.

**Motif analysis**

Transcription factor activity was calculated by chromVAR (v1.10.0), and the JASPAR2020 database (v0.99.10) was used to calculate the positional weight matrix ^5, 6^. The Signac *AddMotifs* function was used to add motif information with full genome sequences for musculus (UCSC v. mm10) ^7^. The Seurat *FindMarker* function was used to find overrepresented motifs ^3^.

**Trajectories analysis**

Monocle3 (v0.2.3.0) was used to build pseudo-temporal trajectories of scATAC-seq or scRNA-seq data ^8^. To convert the Seurat object of scATAC-seq to Monocle3’s cell dataset object (CDS), SeuratWrappers (v0.2.0) was necessary. The Monocle3 *find_gene_modules* function was used to find gene regulated modules determining cell fate from mitosis to meiosis and pheatmap (v1.0.12) was used for visualization.

**RNA velocyto analysis**

RNA velocyto analysis was used to assess transcriptome dynamics. Briefly, we use velocyto.R (v0.6) to perform RNA velocity analysis, and the input is the loom format file generated by velocyto (python tools) ^9^. The state of the cell is judged by evaluating the splicing state of the gene.

**Building** **cis-co-accessible networks (CCANs)**

Cicero (v1.3.4.10) was used to build cis-co-accessible networks (CCANs), that represented the synergistic open region of chromatin, of all scATAC-seq peaks ^10^. Briefly, we initially used the *run_cicero* function to find co-accessibility scores in peaks on whole genomes, then the *generate_ccans* function was adopted to build CCANs.

**Gene ontology (GO) analysis**

The Seurat *FindMarker* function was used to find differentially expressed genes (DEGs) or differentially expressed peaks (DEPs) with threshold *min.pct = 0.25 & log.fcthreshold = 0 & pvalue < 0.05* during meioic initiation ^11^. DEGs or DEPs were processed for GO analysis using clusterProfiler (v3.16.1) ^12^, as well as other gene lists, such as gene regulated modules determining cell fate of mitosis to meiosis, and cell cluster specific peaks.

**Cell-cell communication analysis**

Cell-cell communication plays vital role in regulating the process of biological development. Cellchat (v1.6.1) was adopted to analyze ligand-receptor interactions between different cell types ^13^. We used the gene expression matrix of different germ cell subtypes generated by Seurat as the input file for Cellchat.

**References**

1. Ge W, Wang J-J, Zhang R-Q, Tan S-J, Zhang F-L, Liu W-X*, et al.* Dissecting the initiation of female meiosis in the mouse at single-cell resolution. *Cellular and Molecular Life Sciences* 2021, **78**(2)**:** 695-713.

2. Chuma S, Nakatsuji N. Autonomous transition into meiosis of mouse fetal germ cells in vitro and its inhibition by gp130-mediated signaling. *Developmental biology* 2001, **229**(2)**:** 468-479.

3. Stuart T, Butler A, Hoffman P, Hafemeister C, Papalexi E, Mauck III WM*, et al.* Comprehensive integration of single-cell data. *Cell* 2019, **177**(7)**:** 1888-1902. e1821.

4. Stuart T, Srivastava A, Madad S, Lareau CA, Satija R. Single-cell chromatin state analysis with Signac. *Nature methods* 2021, **18**(11)**:** 1333-1341.

5. Schep AN, Wu B, Buenrostro JD, Greenleaf WJ. chromVAR: inferring transcription-factor-associated accessibility from single-cell epigenomic data. *Nat Methods* 2017, **14**(10)**:** 975-978.

6. Fornes O, Castro-Mondragon JA, Khan A, Van der Lee R, Zhang X, Richmond PA*, et al.* JASPAR 2020: update of the open-access database of transcription factor binding profiles. *Nucleic acids research* 2020, **48**(D1)**:** D87-D92.

7. Stuart T, Srivastava A, Lareau C, Satija R. Multimodal single-cell chromatin analysis with Signac. *bioRxiv* 2020.

8. Cao J, Spielmann M, Qiu X, Huang X, Ibrahim DM, Hill AJ*, et al.* The single-cell transcriptional landscape of mammalian organogenesis. *Nature* 2019, **566**(7745)**:** 496-502.

9. La Manno G, Soldatov R, Zeisel A, Braun E, Hochgerner H, Petukhov V*, et al.* RNA velocity of single cells. *Nature* 2018, **560**(7719)**:** 494-498.

10. Pliner HA, Packer JS, McFaline-Figueroa JL, Cusanovich DA, Daza RM, Aghamirzaie D*, et al.* Cicero predicts cis-regulatory DNA interactions from single-cell chromatin accessibility data. *Molecular cell* 2018, **71**(5)**:** 858-871. e858.

11. Kojima ML, de Rooij DG, Page DC. Amplification of a broad transcriptional program by a common factor triggers the meiotic cell cycle in mice. *Elife* 2019, **8:** e43738.

12. Yu G, Wang LG, Han Y, He QY. clusterProfiler: an R package for comparing biological themes among gene clusters. *OMICS* 2012, **16**(5)**:** 284-287.

13. Jin S, Guerrero-Juarez CF, Zhang L, Chang I, Ramos R, Kuan C-H*, et al.* Inference and analysis of cell-cell communication using CellChat. *Nature communications* 2021, **12**(1)**:** 1-20.
